# Antibacterial Effect of Stainless Steel Surfaces Treated with a Nanotechnological Coating Approved for Food Contact

**DOI:** 10.3390/microorganisms9020248

**Published:** 2021-01-26

**Authors:** Alessandro Di Cerbo, Andrea Mescola, Giuseppe Rosace, Roberta Stocchi, Giacomo Rossi, Andrea Alessandrini, Silvia Preziuso, Antonio Scarano, Stefano Rea, Anna Rita Loschi, Carla Sabia

**Affiliations:** 1 School of Biosciences and Veterinary Medicine, University of Camerino, 62024 Matelica, Italy; roberta.stocchi@unicam.it (R.S.); Giacomo.rossi@unicam.it (G.R.); silvia.preziuso@unicam.it (S.P.); stefano.rea@unicam.it (S.R.); annarita.loschi@unicam.it (A.R.L.); 2 CNR-Nanoscience Institute-S3, 62024 Modena, Italy; andrea.mescola@nano.cnr.it (A.M.); andrea.alessandrini@unimore.it (A.A.); 3 Department of Engineering and Applied Sciences, University of Bergamo, 24044 Dalmine, Italy; giuseppe.rosace@unibg.it; 4 Department of Physics, Informatics e Mathematics, University of Modena and Reggio Emilia, 41125 Modena, Italy; 5 Department of Medical, Oral and Biotechnological Sciences, University of Chieti-Pescara, 66100 Chieti, Italy; ascarano@unich.it; 6 Department of Life Sciences, University of Modena and Reggio Emilia, 41125 Modena, Italy; carla.sabia@unimore.it

**Keywords:** stainless steel, bactericidal effect, roughness, nanotechnological coating, nanoXHAM^®^ D

## Abstract

Stainless steel, widely present in the food industry, is frequently exposed to bacterial colonization with possible consequences on consumers’ health. 288 stainless steel disks with different roughness (0.25, 0.5 and 1 μm) were challenged with four Gram-negative (*Escherichia coli* ATCC 25922, *Salmonella typhimurium* ATCC 1402, *Yersinia enterocolitica* ATCC 9610 and *Pseudomonas aeruginosa* ATCC 27588) and four Gram-positive bacteria (*Staphylococcus aureus* ATCC 6538, *Enterococcus faecalis* ATCC 29212, *Bacillus cereus* ATCC 14579 and *Listeria monocytogenes* NCTT 10888) and underwent three different sanitizing treatments (UVC, alcohol 70% *v/v* and Gold lotion). Moreover, the same procedure was carried out onto the same surfaces after a nanotechnological surface coating (nanoXHAM^®^ D). A significant bactericidal effect was exerted by all of the sanitizing treatments against all bacterial strains regardless of roughness and surface coating. The nanoXHAM^®^ D coating itself induced an overall bactericidal effect as well as in synergy with all sanitizing treatments regardless of roughness. Stainless steel surface roughness is poorly correlated with bacterial adhesion and only sanitizing treatments can exert significant bactericidal effects. Most of sanitizing treatments are toxic and corrosive causing the onset of crevices that are able to facilitate bacterial nesting and growth. This nanotechnological coating can reduce surface adhesion with consequent reduction of bacterial adhesion, nesting, and growth.

## 1. Introduction

Stainless steel is widely present in the food industry as the main component of work surfaces, cookware, pasteurizers, homogenizers, separators, decanters, mixing, process and storage tanks, fittings, valves, pumps, and pipework [1,2,3]. Based on the high resistance to corrosion by acidic or sulfur dioxide-containing foods and cleanability, AISI 316 or 316 L stainless steel should be preferred to AISI 302 or AISI 304 [2,4]. Although naturally passivated by air or other oxidizers, stainless steel needs to undergo additional surface treatments (pickling, electropolishing, and mechanical cleaning) to improve its strength [5].

Nevertheless, microorganisms can hide in crevices present on the stainless steel surface thus escaping cleaning and disinfecting procedures and then become responsible for new re-contaminations during the following food processing [3,6].

New strategies focused on refining stainless steel surface topography and chemistry, to reduce microbial retention and/or attachment, as well as on improving sanitization methods, to fully remove or inactivate viable cells, are therefore necessary.

Surface topography is usually described by the surface roughness (Ra), which is expressed in micrometers and is regarded as a general quality control parameter, since values of less than 0.8 µm indicate a hygienic surface [7].

Studies conducted on surface topography revealed that cleanability and hygienic features of stainless steel finish used in food processing are unaffected by the finish itself [6,8], although electropolished surfaces demonstrated to reduce bacterial attachment, but not retention, with respect to rougher ones [9]. On the other hand, despite increased surface roughness has been shown to strongly affect microbial retention [3,10], several studies underlined the lack of a direct relationship between such parameter and Ra [11,12,13,14,15].

As far as concerns surface chemistry, the selection of the chemical elements (chromium, nickel, manganese, and molybdenum) to be embedded within stainless steel plays a pivotal role in food hygiene, both due to the potential release of such elements from the surface and to the ability to prevent microbial attachment [3,16]. In fact, stainless steel coated with a titanium grid has been shown to favor *Pseudomonas aeruginosa* and *Staphylococcus aureus* binding to the surface but not *Escherichia coli* [3,17,18].

Further, studies conducted on glass surface revealed that attachment and biofilm formation of *Listeria monocytogenes, Salmonella typhimurium, Staphylococcus aureus, Escherichia coli,* and *Pseudomonas aeruginosa* varied among strains and were strongly influenced by hydrophilicity, hydrophobicity, chain length, or chemical functionality [8,19,20,21].

In light of the contrasting behaviors of bacterial strains depending on the substrate, the use of homogeneous and less conducive to retention nanotechnological coatings are necessary to prevent microbial attachment and contamination during food processing.

In the last decade, the deposition of nanotechnological coatings by cathodic arc physical vapor deposition (PVD) [22], cyclonic atmospheric pressure plasma [23], metal injection molding [24], sol-gel process, dip-coating technique [25], and plasma-enhanced chemical vapor deposition (PECVD) [26] gained great importance due to their ability to control metal ions release and enhance hydrophobicity and cleanability. At the same time, the need for new sanitizing treatments able to reduce/inhibit bacterial retention, prevent stainless steel equipment degradation and, in turn, reduce/eliminate food contamination and chemical elements release is also of great relevance [3,27].

The aim of this study, in fact, was to evaluate the potential bacteriostatic/bactericidal efficacy of stainless steel surfaces with different large-scale roughness (0.25, 0.5, and 1 μm) following three different sanitizing treatments [UVC, alcohol 70% *v/v* and gold lotion (GL)] [28,29] against four Gram-negative (*Escherichia coli* ATCC 25922, *Salmonella typhimurium* ATCC 1402, *Yersinia enterocolitica* ATCC 9610 and *Pseudomonas aeruginosa* ATCC 27588) and four Gram-positive bacteria (*Staphylococcus aureus* ATCC 6538, *Enterococcus faecalis* ATCC 29212, *Bacillus cereus* ATCC 14579 and *Listeria monocytogenes* NCTT 10888) before and after deposition of a nanotechnological surface coating named nanoXHAM^®^ D approved for food contact.

## 2. Materials and Methods

### 2.1. The Samples

Large-scale roughness of 288 round-shaped stainless steel (AISI 316, compliant with EN 10204 3.1) disks (SEGAT GIANNI Srl, Gerenzano, Italy) with a 5 cm diameter was analyzed by profilometer (SURFTEST SJ-210, Mitutoyo Italiana S.r.l., Milano, Italy) resulting in three different roughness average values (Ra): 0.25 ± 0.02, 0.5 ± 0.03 and 1 ± 0.06 μm. The disks were then equally divided into three groups of 96 each by roughness average values named R 0.25, R 0.5, and R 1, respectively. After microbiological and microscopic analyses all disks were covered with a surface treatment named nanoXHAM^®^ D.

### 2.2. NanoXHAM Coating

**Figure 1:**
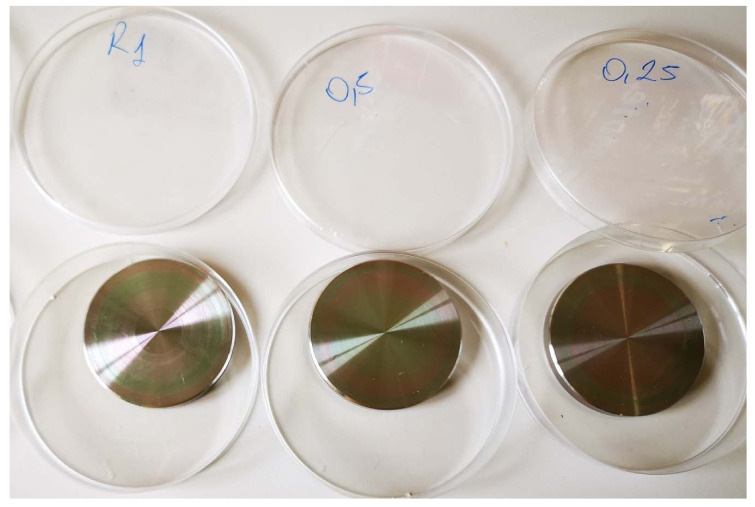
Representative image of stainless steel disks with different roughness, R 0.25, R 0.5, and R 1 μm treated nanoXHAM^®^ D.

NanoXHAM^®^ D [Moma Nanotech s.r.l, Brugherio (MB), Italy] [30] is a coating compliant with regulation 1935/2004/CE and National Sanitation Foundation (NSF) standard 51, and therefore suitable for contact with food products (Figure 1). Moreover, it is also certified as not cytotoxic according to the ISO 10993-5:2009 [30]. It is a transparent thin film of amorphous SiOxCyHz, deposited via PECVD at room temperature [31]. The thickness is about 1 µm, perfectly flexible [26], adherent to the substrate, corrosion, and wear-resistant. The surface of this nanotechnological coating is hydrophobic and the surface tension is about 28 mN/m according to ISO 8296-2003.

### 2.3. FT-IR Spectra

To compare the surface modification introduced by plasma deposition, FT-IR spectra of treated and untreated samples were acquired by means of a Thermo Avatar 370 spectrometer (Thermo Nicolet Corp., Madison, WI, USA), equipped with an attenuated total reflection (ATR) accessory. A diamond crystal was used as an internal reflectance element on the ATR accessory. Spectra were recorded at room temperature, in the range from 4000 to 650 cm^−1^, acquiring 32 scans per set data of 4 cm^−1^ resolution. Two spectra were recorded for each sample.

### 2.4. Bacterial Strains Used in This Study

Reference strains (ATCC—American Type Culture Collection) of *Escherichia coli* ATCC 25922, *Salmonella typhimurium* ATCC 1402, *Yersinia enterocolitica* ATCC 9610, *Pseudomonas aeruginosa* ATCC 27588, *Staphylococcus aureus* ATCC 6538, *Enterococcus faecalis* ATCC 29212, *Bacillus cereus* ATCC 14579 and *Listeria monocytogenes* NCTT 10888 were used in this study. All strains were grown in tryptic soy broth (TSB, bioMérieux, Florence, Italy), incubated at 37 °C for 24 h, and activated by two successive transfers.

### 2.5. Microbiological Analysis

One hundred μL of the overnight cultures of each bacterium were transferred to 10 mL TSB and incubated for 24 h at 37 °C with shaking at 150–250 RPM. Then a further 10-fold dilution in 1 mL of saline 0.9% was done. Cultures were spectrophotometrically measured at 600 nm and the viable cell count was determined by plating onto Tryptic soy agar (TSA).

Suspensions of approximately 10^6^ CFU/mL of each strain were used for inoculating onto stainless steel disks.

One hundred μL of the inoculum were firstly placed at the center of the stainless steel disk and then spread on the whole surface by means of a sterilized spatula, covered by the petri dish lid and placed in the incubator at 37 °C 24 h to render attachment before being processed with sanitizers.

Thirty-six Petri dishes (12 for R 0.25, 12 for R 0.5, and 12 for R 1) containing one stainless steel disk each were used for each bacterial strain. For each roughness, nine out of twelve stainless steel disks underwent one of three different sanitizing treatments i.e., UV (UVC, 253 nm) direct exposure under the hood (70 cm distance), alcohol 70% *v/v,* and gold lotion (GL, Miyauchi Citrus Research Center, Shigoka-Machi Takasaki Gunma, Japan) for 12 h while 3 out of 12 stainless steel disks were not sanitized and worked as negative controls.

One mL of ethanol and GL was applied directly on the disk surface with friction in circular movements for 30” by means of a sterile loop after the inoculum spreading.

A sterile swabbing (Sterile swabs without culture medium, Incofar s.r.l., Modena, Italy) was carried out after 12 h of a challenge with sanitizers by rubbing of the surface at room temperature. Then, the tip of the swab was placed in the sterile swab tube with 1 mL of saline 0.9% and vortexed for one minute. Then we took 0.1 mL from the sterile swab tube and plated them onto the agar plate with a sterile spatula (final concentration 10^−1^).

Serial tenfold dilutions (0.1 mL + 0.9 mL) of re-suspensions were spread onto appropriate agar plates for the viable cell count. The colonies were physically counted on the plate following the incubation at 37 °C for 24 h.

GL is a commercially available natural product made of peels derived from *navel oranges*, *Citrus hassaku, Citrus limon, Citrus natsudaidai, Citrus miyauchi*, and *Satsuma*, with a total content of flavonoids equal to 0.45 mg/mL [32].

### 2.6. Atomic Force Microscopy Analysis

A BioScope I microscope equipped with a Nanoscope IIIA controller (Veeco Metrology, Plainview, NY, USA) was used to acquire AFM topography images. In order to reduce vibrations or movements which negatively affect the tip scan, all the samples were sticked on the underlying substrate.

The BioScope head was then mounted on the top of the samples. AFM imaging was performed in non-contact mode at room temperature, in air; triangular doped silicon cantilevers (Veeco, NTESP) with nominal spring constants between 20 and 80 N/m and a resonance frequency around 270 KHz were used. AFM images post-processing and root mean square (RMS) roughness quantification were obtained by using the free software Gwyddion (v. 2.41).

### 2.7. Environmental Scanning Microscopy Analysis

Morphological analysis of the nanoXHAM^®^ D surface-treated stainless steel disks was performed by scanning electron microscopy (Nova Nano SEM 450, ThermoFisher Scientific, Rodano (MI), Italy) equipped with an energy-dispersive X-ray microanalysis system (X-EDS, QUANTAX-200, Bruker Nano Analytics, Berlin, Germany) using secondary electrons. Each sample was mounted onto a sample stub via double-sided adhesive tape and images were taken at an accelerating voltage of 15 kV.

### 2.8. Statistical Analysis

All the experiments were carried out in triplicate. Data were analyzed using GraphPad Prism 7 software (GraphPad Software, Inc., La Jolla, CA, USA). All data are presented as the means ± standard error of the mean (SEM) and were first checked for normality using the D’Agostino–Pearson normality test. Differences in bacterial growth for each strain at different roughness, both on untreated and nanoXHAM^®^ D-treated disks and after different sanitizing treatments, were analyzed using a two-way analysis of variance (ANOVA) followed by Tukey’s multiple comparison test. The difference among controls of each strain at different roughness, both on untreated and nanoXHAM^®^ D-treated disks, was analyzed using a Kruskal–Wallis test followed by Dunn’s multiple comparison test.

## 3. Results

**Figure 2:**
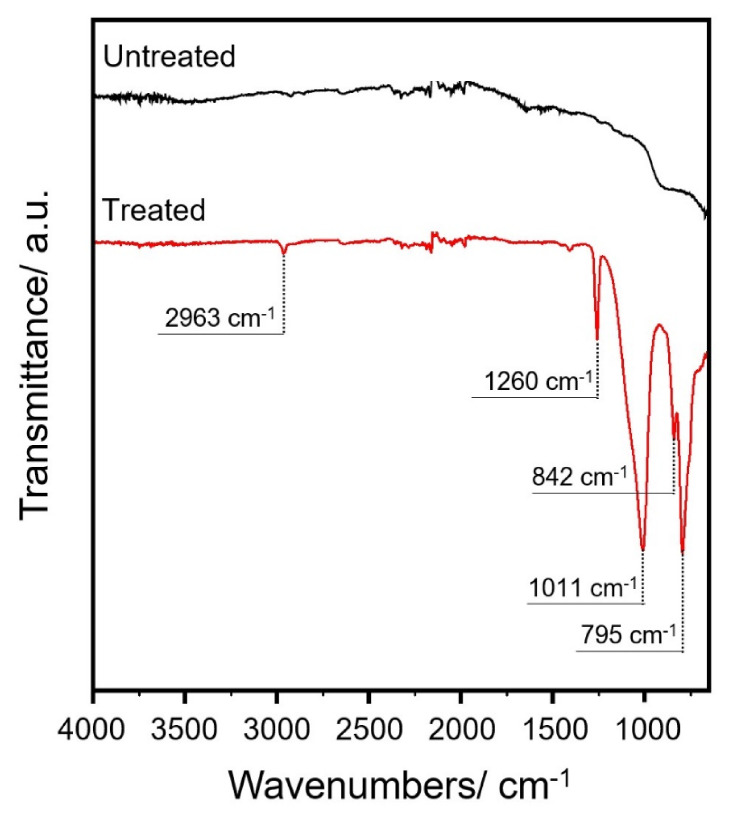
FT-IR spectra (cm^−1^) of untreated stainless steel disks and treated with nanoXHAM^®^ D coating.

During the PECVD treatment, due to inelastic collision with electrons, monomers are dissociated to smaller chemical species or radicals, describing principally via breaking of the Si–O–Si and Si–C bonds. Since plasma deposition modifies the surface of a material only at the micron level, infrared analysis, rather than transmission spectra, is found to be an appropriate technique to characterize the induced chemical modification. The ATR FT-IR spectra of treated and untreated samples, registered in the range between 4000 cm^−1^ and 600 cm^−1^, are shown in Figure 2. The frequencies listed in Table 1 are the main IR absorption peaks detected and assigned to characteristic vibrational modes.

### 3.1. Untreated Stainless steel Samples

**Figure 3:**
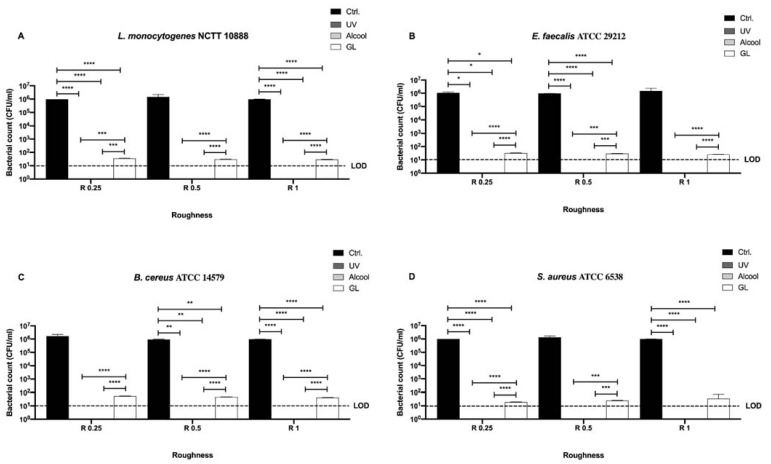
Graphical representation of the antibacterial activity of UV, alcohol 70% *v/v* and GL against Gram-positive bacteria (**A**–**D**) at different AISI 316 stainless steel surface roughness, **** *p* < 0.0001, *** *p* < 0.001, ** *p* < 0.01, * *p* < 0.05; Limit of detection (LOD) is 10^1^ (10E+1).

In Figure 3 differences among the three sanitizing methods (UV, alcohol 70% *v/v,* and GL) and control in different surface roughness (R 0.25, R 0.5 and R 1) against four Gram-positive bacteria (*Staphylococcus aureus* ATCC 6538, *Enterococcus faecalis* ATCC 29212, *Bacillus cereus* ATCC 14579, and *Listeria monocytogenes* NCTT 10888) are represented.

No bacterial count was detectable after UV and alcohol 70% *v/v* treatment for all strains regardless of roughness (Figure 3A–D). As far as concerns GL, a significant decrease in bacterial count was observed after the treatment in R 0.25 for *L. monocytogenes*, *E. faecalis* and *S. aureus* (36 ± 1, 32.67 ± 0.66 and 19.33 ± 0.33 CFU/mL, respectively) when compared with their control (1 × 10^6^, 1.1 ± 0.1 × 10^6^ and 1 × 10^6^ CFU/mL, respectively), **** *p* < 0.0001 and * *p* < 0.05, respectively (Figure 3A,B,D). In R 0.5, only *E. faecalis* and *B. cereus* showed a significant decrease in bacterial count after GL treatment (29.33 ± 0.66 and 47.33 ± 0.33 CFU/mL, respectively) with respect to their control (9.76 ± 0.3 × 10^5^ and 9.46 ± 0.31 × 10^5^ CFU/mL, respectively), **** *p* < 0.0001, ** *p* < 0.01, respectively (Figure 3B,C). As for R 1, *L. monocytogenes, B. cereus* and *S. aureus* showed a significant decrease in bacterial count after GL treatment (29.67 ± 0.33, 41.33 ± 0.66 and 34.67 ± 2.67 CFU/mL, respectively) when compared with their control (9.73 ± 0.14 × 10^5^, 9.93 ± 0.06 × 10^5^ and 9.93 ± 0.06 × 10^5^ CFU/mL, respectively), **** *p* < 0.0001 (Figure 3A,C,D).

**Figure 4:**
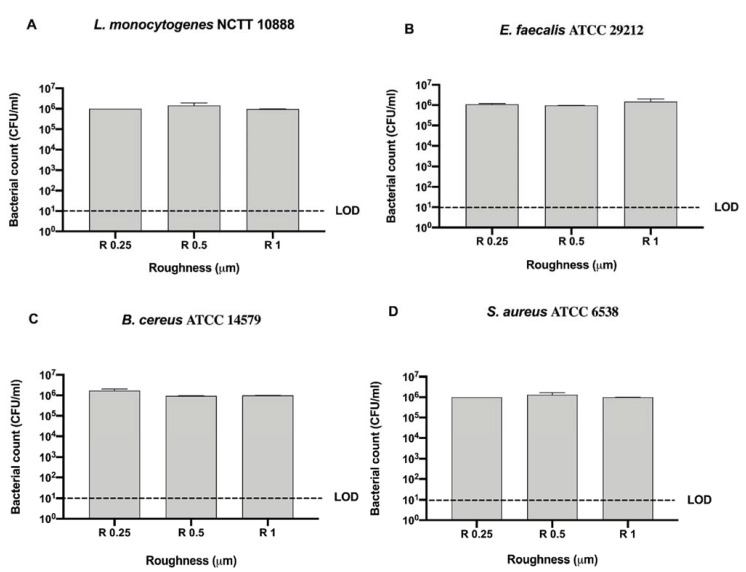
Graphical representation of the antibacterial activity of different AISI 316 stainless steel surface roughness against Gram-positive bacteria (**A–D**); Limit of detection (LOD) is 10^1^ (10E+1).

To better address the antibacterial effect possibly exerted by the surface, we further compared the bacterial count of each strain for each surface roughness without sanitizing methods (Figure 4).

No significant difference was observed among bacterial strains regardless of roughness with respect to the initial inoculum (10^6^ CFU/mL) (Figure 4).

**Figure 5:**
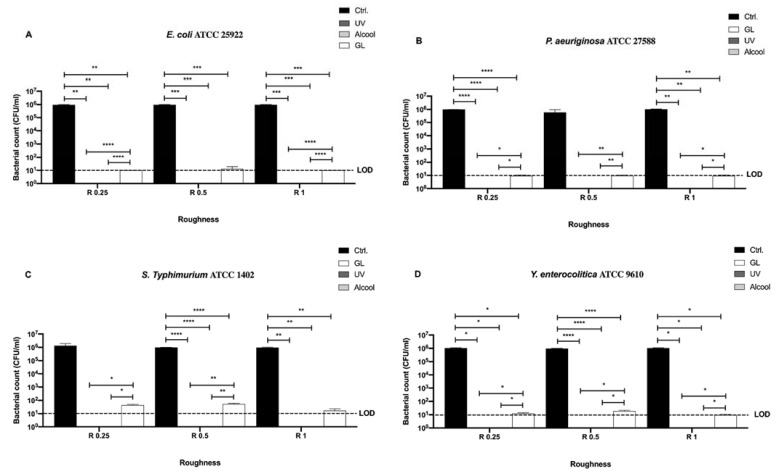
Graphical representation of the antibacterial activity of UV, alcohol 70% *v/v* and GL against Gram-negative bacteria (**A–D**) at different AISI 316 stainless steel surface roughness, **** *p* < 0.0001, *** *p* < 0.001, ** *p* < 0.01, * *p* < 0.05; Limit of detection (LOD) is 10^1^ (10E+1).

We then screened also the Gram-negative bacteria, (*E. coli* ATCC 25922, *S. typhimurium* ATCC 1402, *Y. enterocolitica* ATCC 9610, and *P. aeruginosa* ATCC 27588) and evaluated differences among the three sanitizing methods (UV, alcohol 70% *v/v* and GL) and control in different surface roughness (R 0.25, R 0.5 and R 1 μm) (Figure 5).

As noted for Gram-positive bacteria, no bacterial count was detectable after UV and alcohol 70% *v/v* treatment for all strains regardless of roughness (Figure 5A–D).

A significant decrease in bacterial count was observed in R 0.25 for *E. coli* ATCC 25922, *P. aeruginosa* ATCC 27588 and *Y. enterocolitica* ATCC 9610 (10 ± 0, 9.33 ± 0.66, 12 ± 1 CFU/mL, respectively) when compared with their control (9.43 ± 0.47 × 10^5^, 9.9 ± 0.1 × 10^5^ and 1.06 ± 0.7 × 10^6^ CFU/mL, respectively), ** *p* < 0.01, **** *p* < 0.0001 and * *p* < 0.05, respectively, after GL treatment (Figure 5A,B,D). In R 0.5, *E. coli* ATCC 25922, *S. typhimurium* ATCC 1402 and *Y. enterocolitica* ATCC 9610 showed a significant decrease in bacterial count after GL treatment (13.33 ± 3.33, 53.33 ± 3.33 and 18.67 ± 1.33 CFU/mL, respectively) with respect to their control (9.73 ± 0.26 × 10^5^, 9.83 ± 0.16 × 10^5^ and 9.9 ± 0.1 × 10^5^ CFU/mL, respectively), *** *p* < 0.001 and **** *p* < 0.0001, respectively (Figure 5A,C,D). Interestingly, all strain resulted significantly inhibited in R 1 after GL treatment (10 ± 0, 9.33 ± 0.66, 16.67 ± 0.67 and 9.33 ± 0.67 CFU/mL, respectively), when compared with their control (9.73 ± 0.27 × 10^5^, 1.02 ± 0.37 × 10^6^, 9.5 ± 0.5 × 10^5^ and 1.06 ± 0.7 × 10^6^ CFU/mL, respectively), *** *p* < 0.001, ** *p* < 0.01 and * *p* < 0.05, respectively (Figure 5A–D).

**Figure 6:**
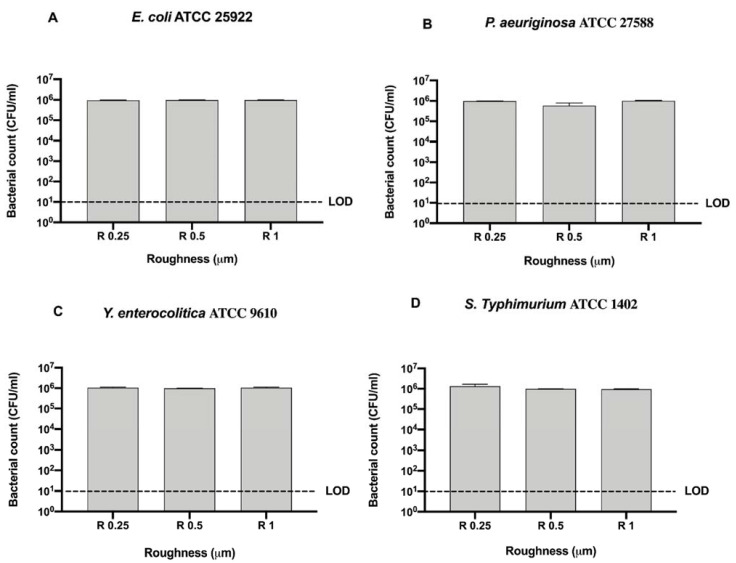
Graphical representation of the antibacterial activity of different AISI 316 stainless steel surface roughness against Gram-negative bacteria (**A–D**); Limit of detection (LOD) is 10^1^ (10E+1).

As for Gram-positive bacteria, we further compared the bacterial count of each Gram-negative strain for each surface roughness without sanitizing methods to better address the possible antibacterial effect exerted by the surface (Figure 6).

No significant difference was observed among bacterial strains regardless of roughness with respect to the initial inoculum (10^6^ CFU/mL) (Figure 6).

### 3.2. NanoXHAM^®^ D-Coated Stainless steel Samples

After microbiological and microscopic analyses all disks were treated with the nanoXHAM^®^ D and both analyses were repeated on treated disks.

**Figure 7:**
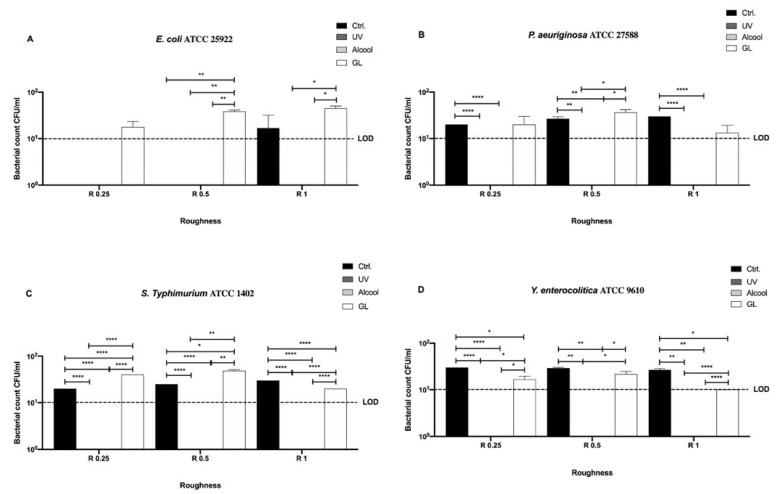
Graphical representation of the antibacterial activity of UV, alcohol 70% *v/v* and GL against Gram-negative bacteria (**A–D**) at different AISI 316 stainless steel surface roughness coated with nanoXHAM^®^ D, **** *p* < 0.0001, ** *p* < 0.01, * *p* < 0.05; Limit of detection (LOD) is 10^1^ (10E+1).

In Figure 7, differences among the three sanitizing methods (UV, alcohol 70% *v/v* and GL) and control at different surface roughness (R 0.25, R 0.5 and R 1 μm) against the same four previous Gram-negative bacteria (*E. coli* ATCC 25922, *S. typhimurium* ATCC 1402, *Y. enterocolitica* ATCC 9610 and *P. aeruginosa* ATCC 27588) are summarized.

Contrary to the uncoated surfaces, a significant mean decrease in the bacterial count of the control was observed for almost all strains (26 ± 0.78 CFU/mL) with respect to the initial inoculum (10^6^ CFU/mL), regardless of roughness (Figure 7B,C,D). Interestingly, no *E. coli* ATCC 25922 count was detected in R 0.25 and R 0.5, while only in R 1 it reached 16.67 ± 8.81 CFU/mL (Figure 7A). Further, as previously reported for the uncoated surfaces, no bacterial count was detectable after UV and alcohol 70% *v/v* treatment for all strains regardless of roughness (Figure 7A–D). As for GL treatment, it showed the same trend for all strains regardless of roughness, in fact, an overall mean significant bacterial count inhibition was observed (27.31 ± 2.25 CFU/mL) when compared with the initial inoculum (10^6^ CFU/mL) (Figure 7A–D).

A significant decrease in bacterial count was observed in R 1 for *S. typhimurium* ATCC 1402 and *Y. enterocolitica* ATCC 9610 (20 ± 0 and 10 ± 0 CFU/mL, respectively) when compared with their control (25 ± 2.23 and 26.67 ± 1.66 CFU/mL, respectively), **** *p* < 0.0001 and * *p* < 0.05, respectively, after GL treatment (Figure 7C,D). However, a significant bacterial count decrease was also observed in R 0.25 for *Y. enterocolitica* ATCC 9610 (18.33 ± 1.05 CFU/mL) with respect to its control (30 ± 0 CFU/mL), * *p* < 0.05 (Figure 7D).

It is noteworthy the significant difference, in terms of bacterial count, between the control (0 CFU/mL) and the GL treatment (29.17 ± 1.16 CFU/mL) observed in R 0.5 for *E. coli* ATCC 25922 (** *p* < 0.01) and the control in R 0.25 and R 0.5 (20 ± 0 and 25 ± 0 CFU/mL, respectively) and the GL treatment (40 ± 0 and 48.33 ± 1.66 CFU/mL, respectively) for *S. typhimurium* ATCC 1402 (**** *p* < 0.0001 and * *p* < 0.05, respectively) (Figure 7A,C).

**Figure 8:**
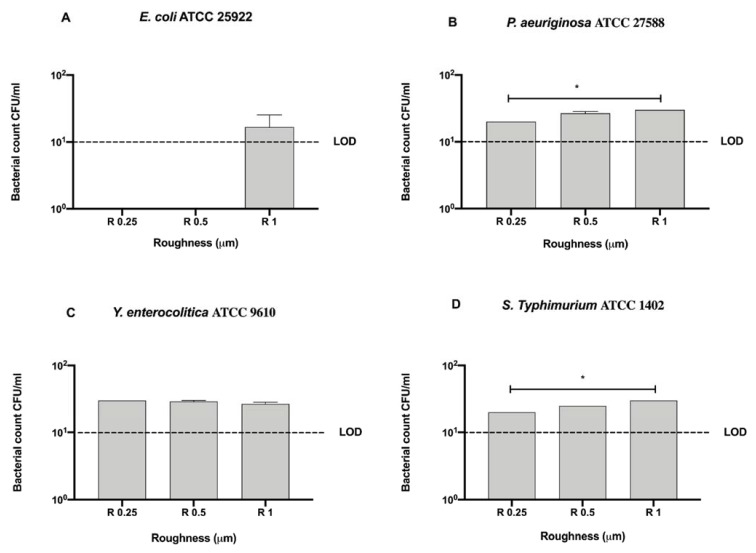
Graphical representation of the antibacterial activity of different AISI 316 stainless steel surface roughness coated with nanoXHAM^®^ D against Gram-negative bacteria (**A–D**), * *p* < 0.05; Limit of detection (LOD) is 10^1^ (10E+1).

To better address the antibacterial effect possibly exerted by the nanoXHAM^®^ D-treatment surface, we further compared the bacterial count of each strain for each surface roughness without the 12-h sanitization with UV, alcohol 70% *v/v,* or GL (Figure 8).

All tested roughness induced a 5-logarithm bacterial count decrease with respect to the initial inoculum (10^6^ CFU/mL), in particular for *E. coli* ATCC 25922 no detectable bacterial count was observed in R 0.25 and R 0.5 (Figure 8). Furtherly, R 0.25 significantly reduced the count of *P. aeruginosa* ATCC 27588 and *S. typhimurium* ATCC 1402 (20 ± 0 and 20 ± 0 CFU/mL, respectively) with respect to R 1 (30 ± 0 and 30 ± 0 CFU/mL, respectively), * *p* < 0.05 (Figure 8B,D).

**Figure 9:**
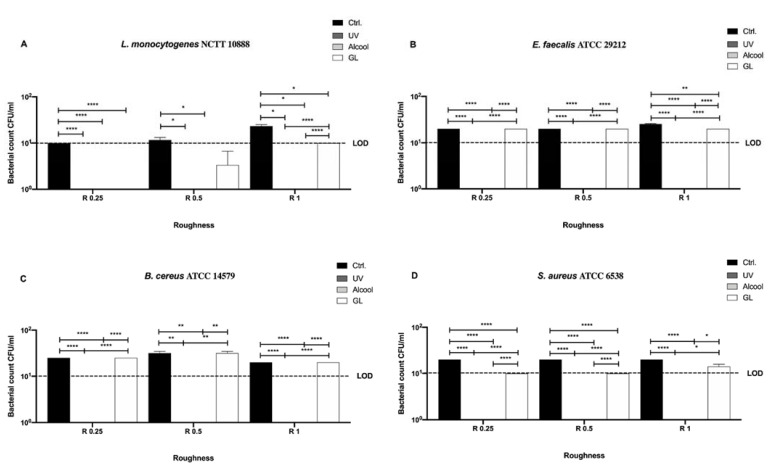
Graphical representation of the antibacterial activity of UV, alcohol 70% *v/v* and GL against Gram-positive bacteria (**A**–**D**) at different AISI 316 stainless steel surface roughness coated with nanoXHAM^®^ D, **** *p* < 0.0001, ** *p* < 0.01, * *p* < 0.05; Limit of detection (LOD) is 10^1^ (10E+1).

As for the untreated disks we also screened Gram-positive bacteria, *L. monocytogenes* NCTT 10888, *E. faecalis* ATCC 29212, *B. cereus* ATCC 14579, and *S. aureus* ATCC 6538, on the nanoXHAM^®^ D-surface treatment (Figure 9).

As noticed for Gram-negative bacteria, no bacterial count was detected after UV and alcohol 70% *v/v* treatment regardless of roughness (Figure 9A–D).

Further, a significant mean decrease in bacterial count of the control was observed for all strains (20.58 ± 0.95 CFU/mL) with respect to the initial inoculum (10^6^ CFU/mL), regardless of roughness (Figure 9A–D). Dealing with GL sanitizing method, a significant decrease was observed in R 1 for *L. monocytogenes* NCTT 10888, *E. faecalis* ATCC 29212 (10 ± 0 and 20 ± 0 CFU/mL, respectively) when compared with their control (23.33 ± 1.67 and 25.33 ± 0.33 CFU/mL, respectively), * p < 0.05 and ** p < 0.01, respectively (Figure 9A,B). Moreover, a significant decrease was also reported in R 0.25 and R 0.5 for *S. aureus* ATCC 6538 (10 ± 0 CFU/mL) with respect to the control (20 ± 0 CFU/mL), **** p < 0.0001 (Figure 9D). As for *E. faecalis* ATCC 29212 and *B. cereus* ATCC 14579, a similar bacterial count decrease was observed in R 0.25 (20 ± 0 CFU/mL) and R 0.5 (20 ± 0 CFU/mL), respectively, and in R 1 only for *B. cereus* ATCC 14579 (20 ± 0 CFU/mL).

**Figure 10:**
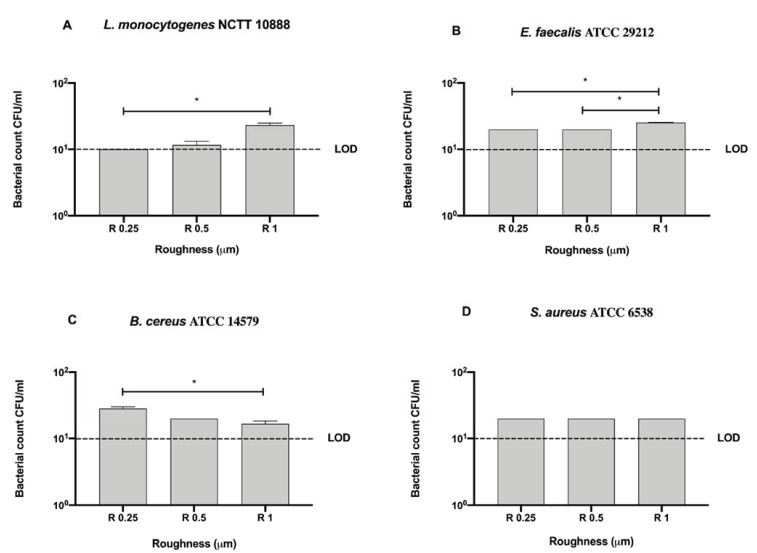
Graphical representation of the antibacterial activity of different surface roughness on nanoXHAM^®^ D-treated disks against Gram-positive bacteria (**A–D**), * *p* < 0.05; Limit of detection (LOD) is 10^1^ (10E+1).

As noted for Gram-negative strains, all tested roughness induced a 5-logarythm bacterial count decrease with respect to the initial inoculum (10^6^ CFU/mL) (Figure 10). Both *L. monocytogenes* NCTT 10888 (10 ± 0 CFU/mL) and *E. faecalis* ATCC 29212 (20 ± 0 CFU/mL) count resulted significantly reduced in R 0.25 with respect to R 1 (23.33 ± 1.66 and 25.33 ± 0.33 CFU/mL, respectively), * *p* < 0.05 (Figure 10A,B).

Further, a significant decrease in bacterial count was also observed for *E. faecalis* ATCC 29212 in R 0.5 (20 ± 0 CFU/mL) with respect to R 1 (25.33 ± 0.33 CFU/mL), * *p* < 0.05 (Figure 10B). Conversely, a significant decrease in bacterial count was also observed for *B. cereus* ATCC 14579 in R 1 (20 ± 0 CFU/mL) with respect to R 0.25 (25 ± 0 CFU/mL), * *p* < 0.05 (Figure 10C).

**Figure 11:**
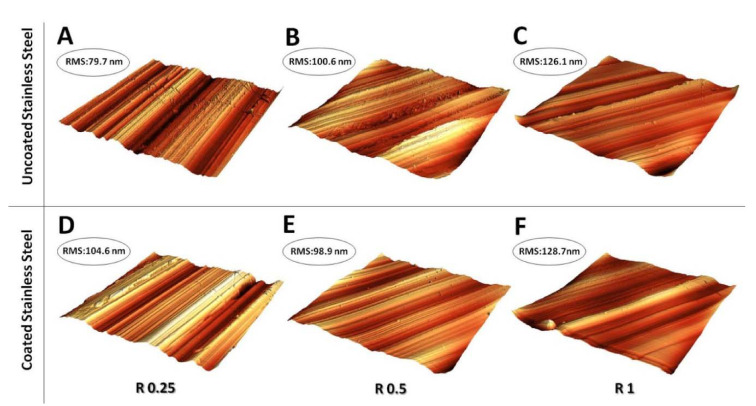
Small range (30 × 30 μm) 3D AFM topographic reconstruction of stainless steel disk surface before (**A**–**C**) and after (**D**–**F**) nanoXHAM^®^ D surface treatment. The relative large-scale roughness values (R 0.25, R 0.5 and R 1) are indicated at the bottom of panels. RMS roughness values correspondent to each scan are reported in the top left corner.

Stainless steel surfaces exhibiting different roughness were also examined in a small range by AFM before and after the nanoXHAM^®^ D surface treatment (Figure 11).

The small range RMS roughness analysis reveals for the uncoated stainless steel surface (Figure 11A–C) a tendency similar to that detected on a larger scale, namely with a progressive roughness increasing from R 0.25 to R 1. Interestingly, after the nanoXHAM^®^ D surface treatment (panels C, D, and F) RMS roughness appears to be almost constant. This represents a clear indication that the coating deposition procedure does not affect the initial surface roughness and results in a homogeneous covering layer regardless of the initial values of roughness.

**Figure 12:**
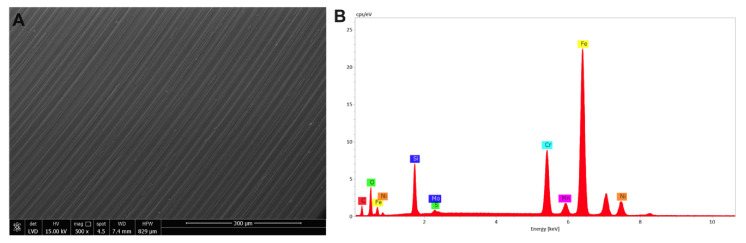
Environmental scanning microscopy morphological analysis on a (**A**) nanoXHAM^®^ D-treated stainless steel disk surface observed at 300 μm along with its (**B**) X-EDS microanalysis.

As observed for the AFM, ESEM images acquired on nanoXHAM^®^ D-treated stainless steel disks confirmed the presence of slanting lines over the surface as well as the presence of a homogeneous layer made of film amorphous SiOxCyHz (Figure 12A). The X-EDS microanalysis confirmed the presence of the aforementioned elements (Figure 12B).

## 4. Discussion

In this study, we reported the significant bactericidal effect exerted by all the three sanitizing treatments tested against all bacterial strains, with respect to the initial inoculum (10^6^ CFU/mL), regardless of roughness and surface coating. At the same time, it is worth pointing out the more significant effect exerted by UV and alcohol 70% *v/v* treatments as no viable bacterial cells were detected under the same conditions.

Results concerning the lack of differences among bacterial strain growth on uncoated stainless steel disks, regardless of surface roughness, are in agreement with other literature reports [6,15,37,38].

Conversely, the nanoXHAM^®^ D induced some results that were as interesting as conflicting. In fact, if on one hand a bactericidal effect was also observed both in the control and after all sanitizing treatments regardless of roughness, a quite puzzling, but not significant, increase in bacterial count was observed after GL treatment, with respect to the control, in most Gram-negative bacteria.

Although we did not perform any punch-through experiments to investigate the possible presence of forces that could take place at the surface/bacterial membrane interface, we believe that such differences might be ascribed to a different pH gradient created by GL and to the incubation temperature (37 °C).

In fact, we previously demonstrated that the physical status of supported (on silicon) lipid bilayers, which share the same physicochemical features with Gram-negative bacteria, can be strongly influenced by environmental conditions such as pH and temperature [39,40]. By means of the AFM, we observed that a pH value of 3.5 was able to promote a bilayer stiffening through a surface charge and electrostatic free energy modification with a consequent formation of small holes, which is the first step towards bacterial cell disruption, although this process is slowed at temperatures higher than 22 °C.

Being GL pH value close to 4.3, it is reasonable to hypothesize a process similar to that described above and therefore the slight increase in count observed in Gram-negative bacteria is due to a delayed death process induced by pH and temperature.

Thanks to the versatility of AFM, some authors also underlined the ease of removal of two different kinds of bacteria (*S. aureus* and *P. aeruginosa*) depending on the surface features [41]. They reported the ease of removal of *S. aureus* from smooth surfaces and of *P. aeruginosa* from 0.5μm surfaces, addressing such difference to the different cell/surface contact area. Indeed, cocci had a smaller cell/surface contact area on smooth surfaces with respect to rods. However, this aspect was observed only for *E. coli* ATCC 25922 in R 0.25 and R 0.5 of nanoXHAM^®^ D-treated control disks.

As far as concerns the comparison among nanoXHAM^®^ D-treated control disks, the growth of two Gram-negative (*S. typhimurium* ATCC 1402 and *P. aeruginosa* ATCC 27588) and of two Gram-positive bacteria (*L. monocytogenes* NCTT 10888 and *E. faecalis* ATCC 29212) resulted to be significantly reduced in R 0.25 with respect to R 0.5 and in particular to R 1.

Moreover, *E. faecalis* ATCC 29212 growth was also significantly reduced in R 0.5 with respect to R 1 while *B. cereus* ATCC 14579 growth was reduced in R 1 with respect to R 0.5 and in particular R 0.25.

It is worth noting that cytotoxicity assays conducted on nanoXHAM^®^ D-coated stainless steel surfaces revealed the lack of any cytotoxic effect of the coating and, therefore, the significant bacterial count decrease observed on all nanoXHAM^®^ D-coated disks might be ascribed to a synergistic effect of low bacterial attachment forces and an increased hydrophobicity of the treatment.

Moreover, FT-IR–ATR, SEM, and EDX elemental analysis measurements confirmed that the organic silicon thin film (nanoXHAM^®^ D) was successfully deposited on stainless steel disks. In particular, the ATR technique of treated samples, compared to the steel sample reference, showed absorption characteristics assigned to C-H stretching, Si–(CH3)x bending, Si-O stretching and Si-O bending modes, respectively.

We also confirmed previously achieved results concerning the potential use of a new, natural, and non-corrosive sanitizing product (GL) to achieve significant bactericidal effect against all tested bacterial strains [29,42]. In particular, GL is rich in flavonoids, which are natural compounds that have been extensively studied for their antibacterial properties [29,43,44,45], and can be considered a valuable alternative candidate to commercially available sanitizing products for stainless steel such as iodine, biguanide, quaternary ammonium compounds, peracetic acid and sodium hypochlorite that showed evident corrosive action [46].

## 5. Conclusions

We can conclude that untreated stainless steel surface roughness is poorly correlated with bacterial adhesion and only sanitizing treatments can exert significant bactericidal effects. Unfortunately, most sanitizing treatments are toxic and corrosive in the long run causing the onset of crevices that are able to facilitate bacterial nesting and growth.

With the advent of this new nanotechnological coating i.e., nanoXHAM^®^ D, it has been possible to change the surface physicochemical characteristics obtaining an overall bactericidal effect possibly due to a synergistic effect of low bacterial attachment forces, increased hydrophobicity, and less toxic and corrosive sanitizing treatments such as UV ethanol and GL.

It is therefore necessary now to accurately undertake time-course experiments with such sanitizing treatments to better exploit the potential of nanoXHAM^®^ D and achieve the best result within the shortest time to fulfill food industry regimes.

## Figures and Tables

**Table 1:** Main vibration modes ascribable to the nanoXHAM^®^ D applied on the steel sample.

Frequencies (cm^−1^)			Vibrational Modes
On Steel Sample	From Literature	Reference	
2963	2980–2800	[33]	n (C-H)
1260	1260	[33]	d (Si–(CH_3_)_x_)
1011	1010–1090	[34]	n (Si-O)
842	840	[35]	r (C-H)
795	800	[36]	d (Si–O)

## Data Availability

The data presented in this study are available on request from the corresponding author.
